# Depression and risk of transformation of episodic to chronic migraine

**DOI:** 10.1007/s10194-012-0479-9

**Published:** 2012-09-25

**Authors:** Sait Ashina, Daniel Serrano, Richard B. Lipton, Morris Maizels, Aubrey N. Manack, Catherine C. Turkel, Michael L. Reed, Dawn C. Buse

**Affiliations:** 1 Department of Neurology, Albert Einstein College of Medicine, Bronx, NY USA; 2 Department of Pain Medicine and Palliative Care, Albert Einstein College of Medicine, Beth Israel Medical Center, New York, NY USA; 3 Vedanta Research, Chapel Hill, NC USA; 4 Montefiore Headache Center, 1575 Blondell Avenue, Suite 225, Bronx, NY 10461 USA; 5 Blue Ridge Headache Center, Asheville, NC USA; 6 Allergan, Inc., Irvine, CA USA

**Keywords:** Depression, Chronic migraine, Epidemiology, Transformation, Migraine, Risk factors

## Abstract

The aim of this study was to assess the role of depression as a predictor of new onset of chronic migraine (CM) among persons with episodic migraine (EM). The American Migraine Prevalence and Prevention (AMPP) study followed 24,000 persons with severe headache identified in 2004. Using random-effects logistic regression, we modeled the probability that persons with EM in 2005 or 2006 would develop CM in the subsequent year. Depression was assessed in two ways, using a validated questionnaire (PHQ-9 score ≥15) and based on self-reported medical diagnosis. Analyses were adjusted for multiple covariates including sociodemographics, body mass index, headache pain intensity, headache frequency, migraine symptom severity, cutaneous allodynia, acute medication overuse, anti-depressant use and anxiety. Of 6,657 participants with EM in 2005, 160 (2.4 %) developed CM in 2006. Of 6,852 participants with EM in 2006, 144 (2.2 %) developed CM in 2007. In fully adjusted models, PHQ-9 defined depression was a significant predictor of CM onset [odds ratio (OR) = 1.65, 95 % CI 1.12–2.45]. There was a depression-dose effect; relative to participants with no depression or mild depression, those with moderate (OR = 1.77, 95 % CI 1.25–2.52), moderately severe (OR = 2.35, 95 % CI 1.53–3.62), and severe depression (OR = 2.53, 95 % CI 1.52–4.21) were at increased risk for the onset of CM. Among persons with EM, depression was associated with an increased risk of CM after adjusting for sociodemographic variables and headache characteristics. Depression preceded the onset of CM and risk increased with depression severity suggesting a potentially causal role though reverse causality cannot be excluded.

**Electronic supplementary material:**

The online version of this article (doi:10.1007/s10194-012-0479-9) contains supplementary material, which is available to authorized users.

## Introduction

Migraine, a chronic disorder with episodic attacks, can be subtyped based on attack frequency into episodic migraine (EM) or chronic migraine (CM) [1]. In EM, headache attacks occur on <15 days/month. In CM, considered a complication of EM, headache attacks occur on ≥15 days/month for at least 3 months [1, 2]. The population prevalence of CM ranges from 1.3 to 2.4 % [3].

The process of developing CM from EM, sometimes termed “transformation” or “progression” occurs in approximately 2 % of persons with EM annually [4, 5]. Epidemiologic studies and clinical observations support a transition model for migraine [6]. In this model, transformation is associated with various modifiable and unmodifiable risk factors [4, 5]. Modifiable risk factors may provide targets for future preventive interventions, designed to reduce new onset of CM.

Both clinic and population-based studies have demonstrated that, compared to EM, CM is associated with greater migraine-related disability [7, 8], reduced quality of life [9], increased resource utilization [8], and increased medical and psychiatric comorbidities including depression [10]. Epidemiologic studies estimate lifetime prevalence rates of major depression of 5–17 % in the general US population [11]. Migraine and depression are highly comorbid and share a bidirectional relationship [12]. The effect of depression on the new onset of CM in episodic migraineurs has not been examined in longitudinal, population-based studies.

Herein, we assess the role of depression as a predictor of the new onset of CM in individuals with EM using data from the AMPP study database. Our estimates of the depression effect are adjusted for a comprehensive set of covariates to better assess the contribution of depression after taking potential confounders into account.

## Methods

### Study survey and population

The AMPP study is a two-phase, longitudinal, population-based survey of headache epidemiology. Sampling methods and design have been described elsewhere [13]. Briefly, in phase 1, a self-administered headache screening survey was mailed in June 2004 to a stratified random sample of 120,000 US households. The 2004 sample yielded data for 162,756 household members aged 12 and older with gender-equivalent response rates. A random sample of 24,000 adults (age 18 and older) was selected from the 30,721 respondents reporting at least one severe headache in the past year in the 2004 screener. This random sample was enrolled in phase 2: an ongoing longitudinal follow-up study. To be included in the current study, respondents had to meet criteria for EM in 2005 and return valid questionnaires in 2005, 2006, and 2007. The study was approved by the Albert Einstein College of Medicine, Institutional Review Board.

### Headache diagnosis and case definitions

The headache module of the AMPP study includes items that assess migraine features according to ICHD-2 criteria [1]. The module has a sensitivity of 100 % and specificity of 82 % for migraine diagnosis [14], and sensitivity of 93 % and specificity of 85 % for CM diagnosis [15]. EM was defined as headache occurring <15 days/month on average over the preceding 3 months and fulfilling ICHD-2 criteria for migraine. CM was defined as having headache on ≥15 days/month averaged over the preceding 3 months and meeting ICHD-2 criteria for migraine. The criteria used are a variation from the ICHD-2R definition of CM [2]. The new onset of CM (i.e., transformation) was defined as: (1) having EM in 2005 and developing CM in 2006 and/or (2) having EM in 2006 and developing CM in 2007. The reference groups for the two transformation events were those who did not develop CM within the time interval of interest and who never met criteria for CM in the years 2005–2007.

### Assessment of independent variables

Depression was measured in two ways: using a validated questionnaire (the Patient Health Questionnaire-depression module (PHQ-9) [16]) and based on self-reported physician diagnosis (SRPD-Depression). The PHQ-9 provides a validated measure of current Major Depressive Disorder based on DSM-IV criteria [16]. The PHQ-9 assesses symptoms and functional impairment over the preceding 2 weeks and contains nine items with four frequency response options (scored as 0, 1, 2, and 3). A sum score is used to categorize participants into four depression categories: none/minimal (0–4), mild (5–9), moderate (10–14), moderately severe (15–19), and severe (20–27). A cut score of 15 was used to define the dichotomous depression variable. In models assessing the dose response for depression, we combined none/minimal and mild into the reference group against which moderate, moderately severe, and severe were separately contrasted. The SRPD-depression item assessed rates of ever being diagnosed with major depression by a physician.

A variety of covariate adjustments were included to assess the robustness of the depression effect and unique contribution of the adjusting effects. Covariates included were selected based on theoretical relevance and evidence in the literature linking them to the emergence of CM. Variables employed for adjustment included sociodemographic features, headache features (including attack frequency, pain intensity, symptom severity and allodynia), comorbid health conditions such as anxiety as well as medication use.

Sociodemographic data such as age, gender, weight, insurance, and income were obtained via self-report. Body mass index (BMI) was calculated using the standard formula based on self-reported height and weight.

Headache features included a measure of average pain intensity on a scale of 0–10 with 0 indicating no pain and 10 indicating the most severe pain possible [17]. Average pain responses were dichotomized at scores ≥4, defining moderate to severe pain. In addition, monthly headache frequency in years preceding transformation to CM was assessed by self-report. Monthly headache frequency estimates were obtained by averaging self-reported 3 month frequency values.

Migraine symptom severity was obtained from the sum of the seven ICHD-2 migraine-defining features for migraine without aura (unilateral pain, pulsatile pain quality, pain intensity and pain increased by routine physical activity as well as nausea, photophobia, and phonophobia) plus an item assessing visual aura. The primary symptom items are coded to have the following response options: never/rarely (0), less than half the time (1), and half the time or more (2). The visual aura item was coded as no (0) or yes (2). The sum of these items produces the migraine symptom severity score, with values ranging from 0 to 16.

Cutaneous allodynia was assessed with the 12-item Allodynia Symptom Checklist, which includes questions about the frequency of various allodynia symptoms associated with headaches [18]. Total scores range from 0 to 24 with scores ≥3 defining the presence of allodynia.

Anxiety (SRPD-anxiety), a health condition comorbid with depression, was addressed using self-reported medical diagnosis, employing the same open recall period as that of depression. Weekly alcohol consumption and smoking behavior, two additional health conditions comorbid with depression were assessed by self-report. Because alcohol and smoking measures were only available in the 2006 AMPP battery, they were not included in longitudinal analyses.

Two classes of medication use, use of anti-depressants and medication overuse, were also examined because each may contribute to confounding of the link between depression and transformation to CM. Our measure of anti-depressant use was based on self-reported current use of any anti-depressant compounds (including duloxetine, venlafaxine, amitriptyline, nortriptyline, paroxetine, fluoxetine, sertraline). Medication overuse was assessed using the acute medication module, which evaluated self-reported use of simple analgesic compounds, triptans, ergotamines, opioids, and other compounds. Respondents met criteria for our medication overuse definition if they used triptans, opioids, or ergotamines 10 or more days per month, or used simple analgesics 15 or more days per month.

### Statistical analyses

Analyses were performed using SAS Version 9.2 (SAS Institute Inc., Cary, NC, USA). We used a nominal alpha level of 0.05 for statistical testing. Analysis proceeded in two stages. In the first stage, descriptive analyses were conducted characterizing unadjusted differences between those who developed CM and those who did not from 2005 to 2006 and then for the 2006–2007 interval. Contrasts for normally distributed variables (e.g., age, BMI) were based on *t* tests. For binary variables (e.g., gender, and allodynia), contrasts were based on Chi-square tests of the logistic regression odds ratio (OR). For ordered categorical variables (e.g., income and PHQ-9 categories), contrasts were based on the Chi-square test associated with the cumulative logistic OR. Because headache frequency and weekly alcohol use were count variables, inference for the mean comparisons was based on Negative Binomial models.

In the second stage of analysis, two-stage logistic transition models were used to model the new onset of CM in 2006 and 2007 as a function of covariates in the preceding year. The GLIMMIX Procedure for generalized linear mixed models was used for estimation. We parameterized the model using a binomial response distribution, logit inverse link function, and constant subject-specific random effect (detailed information on the model, estimation procedure, and code are available upon request). CM development could be reported in 2006 or in 2007. As depression is a risk factor whose status changes over time, we used a lagged predictor approach. Specifically, odds of developing CM in any subsequent year were modeled from predictor values in the preceding year. Sociodemographics, such as gender and income, were fixed within subject, thus lags were not employed. Other effects, including, but not limited to, age, BMI, headache frequency, allodynia, and depression status were lagged.

We examined a series of four models, with the depression effect included in each. Model 1 focused on adjustment for sociodemographic variables including age, gender, income, insurance, and BMI (both a linear and quadratic trend). Model 2 was fully adjusted by the addition of the lagged cutaneous allodynia effect and lagged effects of SRPD-anxiety, average headache pain intensity, headache frequency, and migraine symptom severity, use of anti-depressant medication and medication overuse.

To elucidate the relationship between depression and CM onset, two additional models were fit. Model 3 examined a dose–response effect of depression across categories (moderate, moderately severe, and severe) relative to participants with none/mild levels of depressive symptoms adjusting for sociodemographics, cutaneous allodynia, SRPD-anxiety, and migraine pain intensity. Model 4 included an additional adjustment for monthly headache day frequency and SRPD-depression in the years preceding transformation to CM.

Because analyses were restricted to subjects contributing in each year from 2005 to 2007, missing data were not caused by attrition, but by nonresponse to specific predictors and covariates. PROC GLIMMIX does not permit missing values on predictors, therefore, we implicitly assume that covariate nonresponse was missing completely at random [19]. Predictor-specific missing values were consistent across all adjusted models and therefore a common sample size for events and trials exists for models contained in Tables 2 and 3 as well as all web tables.

## Results

**Fig. 1:**
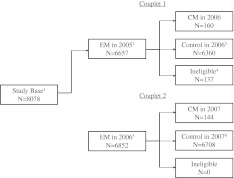
Disposition of subjects during the study. *1* The study base: respondents who provided data in 2005, 2006, 2007 and EM in either 2005 or 2006 or both. *2* 1,921 participants with EM in 2006 but not 2005. *3* Of the 6,360 controls in 2006, 5,312 meet ICHD-2 criteria for EM and 1,048 has other outcomes including PM and ETTH. *4* 137 subjects with other outcomes were excluded because they developed CM in 2008. *5* The EM subjects in 2006 included 5,331 with EM in 2005 and 2006, and 1,921 subjects with EM in 2006 but not 2005 for a total of 6,852. *6* The 6,708 control subjects in 2007 included 5,212 with EM, 759 with PM, 691 with ETTH and any other outcomes. *EM* episodic migraine, *CM* chronic migraine

**Fig. 2:**
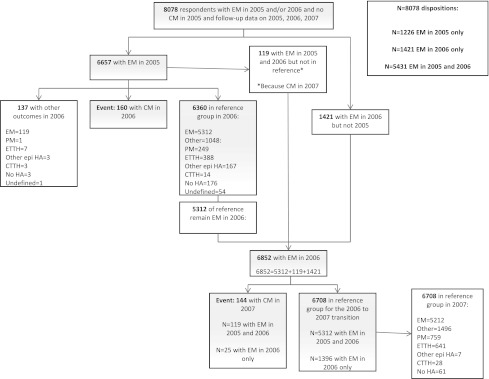
The full disposition of the analysis sample. *EM* episodic migraine, *CM* chronic migraine, *PM* probable migraine, *ETTH* episodic tension-type headache, *CTTH* chronic tension-type headache, *epi* episodic, *HA* headache

The study sample was composed of 8,078 persons who had EM in 2005 or 2006 or both, did not have CM in 2005, and provided follow-up data in 2006, and 2007 (Fig. 1). There were 6,657 eligible participants with EM in 2005, and 160 of these developed CM in 2006. There were 6,852 eligible participants with EM in 2006. Of these, 144 developed CM in 2007. The full disposition of the analysis sample is presented in Fig. 2. Some individuals, not eligible for analysis for the 2005–2006 couplet due to missing data were eligible for the 2006–2007 couplet.

We compared the baseline features in those with EM in 2005 based on outcome status [CM vs. other eligible outcomes (control group)] in 2006 (Table 1). In unadjusted analyses, persons with CM at follow-up in 2006 had lower income levels, and higher rates of cutaneous allodynia, SRPD-anxiety, pain intensity, headache frequency, migraine symptom severity, anti-depressant use, medication overuse, PHQ-9 total scores, PHQ-9 categories, and SRPD-depression rates, than those who did not develop CM at follow-up. Findings were similar for the 2006–2007 transformation event, where 2006 variables were employed as predictors for transformation to CM in 2007 (Table 1).

**Table 1:** Characteristics of persons with EM based on outcome the next year (CM vs. other outcomes) for 2005/2006 and 2006/2007

Predictors in persons with EM for outcomes the subsequent year	Predictor values (2005)			Predictor values (2006)		
	CM in 2006 (*N* = 160)	Control in 2006 (*N* = 6,360)	*p* value (2005–2006)	CM in 2007 (*N* = 144)	Control in 2007 (*N* = 6,708)	*p* value (2006–2007)
Age	47.3 (12.0)	48.4 (13.0)	0.30	48.1 (12.9)	48.7 (12.9)	0.59
Gender (female)	130 (81.3)	5,164 (81.2)	0.99	116 (87.9)	5,106 (82.0)	0.08
Income						
<$22,500	49 (30.6)	1,571 (24.7)	−	56 (42.4)	1,506 (24.2)	−
$22,500–$39,999	34 (21.3)	1,256 (19.8)	−	33 (25.0)	1,220 (19.6)	−
$40,000–$59,999	25 (15.6)	1,227 (19.3)	−	14 (10.6)	1,227 (19.7)	−
$60,000–$89,999	35 (21.9)	1,154 (18.1)	−	15 (11.4)	1,141 (18.3)	−
≥$90,000	17 (10.6)	1,152 (18.1)	0.03	14 (10.6)	1,134 (18.2)	≤0.001
Marital status (married)	4,067 (63.95)	101 (63.13)	0.831	4,291 (63.97)	80 (55.56)	0.039
Race (Caucasian)	5,497 (88.11)	148 (94.27)	0.021	5,417 (88.64)	119 (90.15)	0.589
BMI	30.3 (10.0)	29.2 (7.7)	0.08	29.5 (8.3)	29.4 (7.7)	0.9
SRPD-hypertension	1,851 (29.10)	39 (24.38)	0.194	2,121 (31.62)	54 (37.50)	0.135
SRPD-diabetes	688 (10.82)	21 (13.13)	0.355	–	–	–
Cutaneous allodynia	109 (68.1)	3,436 (54.0)	≤0.001	95 (66.0)	3,761 (56.1)	0.02
SRPD-anxiety	55 (34.4)	1,157 (18.2)	≤0.001	33 (25.0)	1,122 (18.0)	0.04
Pain intensity	154 (98.1)	5,834 (93.4)	0.03	136 (100)	6,281 (99.2)	1.0^a^
Headache frequency (days/month)	6.8 (4.3)	2.6 (2.8)	≤0.001	6.1 (4.4)	2.5 (2.8)	≤0.001
Migraine symptom score	12.6 (2.9)	11.3 (3.7)	≤0.001	12.7 (3.1)	11.6 (3.5)	≤0.001
Current smoking	–	–	–	1,116 (16.64)	31 (21.53)	0.121
Weekly alcohol use	–	–	–	2.93 (5.35)	2.85 (6.59)	0.879
Use of anti-depressants	1,100 (17.30)	53 (33.13)	≤0.001	1,116 (16.64)	33 (22.92)	0.047
Medication overuse	700 (11.01)	47 (29.38)	≤0.001	606 (9.03)	20 (13.89)	0.048
Depression (PHQ-9 total score)	8.76 (6.2)	5.74 (5.4)	≤0.001	8.1 (6.8)	5.3 (5.3)	≤0.001
Depression (PHQ-9 categories)						
None/mild	96 (60.8)	5,043 (80.7)		94 (65.7)	5,472 (82.6)	
Moderate	31 (19.6)	678 (10.9)		22 (15.4)	667 (10.1)	
Moderately severe	20 (12.7)	334 (5.3)		13 (9.1)	289 (4.4)	
Severe	11 (6.9)	195 (15.9)	≤0.001	14 (9.8)	200 (3.0)	≤0.001
SRPD-depression	70 (43.8)	1,519 (23.9)	≤0.001	63 (43.75)	1,992 (29.7)	≤0.001

Mean (standard deviation) was calculated for age, income, BMI, average headache pain rating, headache frequency, migraine symptom score, weekly alcohol use and PHQ-9 total score. Proportion (percentage) was calculated was calculated for female gender, income groups, marital status, race, hypertension, diabetes, cutaneous allodynia, SRPD-anxiety, current smoking, use of anti-depressants, medication overuse, PHQ-9 categories, and SRPD-depression SRPD: self-reported physician diagnosed. Variables: age (continuous), gender (binary, reference = male), marital status (binary, reference = unmarried), race (binary, reference = non-Caucasian), BMI (continuous), SRPD-hypertension (binary, reference = no hypertension), SRPD-diabetes (binary, reference = no diabetes), allodynia (binary, diagnosis defined as score >3), SRPD-anxiety (binary, with no SRPD-anxiety endorsement as reference), pain intensity (binary, defined as score >4), headache frequency (treated as a count of headache days/month), migraine symptom score (continuous), current smoking (binary, reference = not currently smoking), weekly alcohol use (count variable), use of anti-depressants (binary, reference = no use), medication overuse proxy (binary, reference = no medication overuse), PHQ-9 total score (continuous), PHQ-9 categories (categorical), SRPD-depression (binary, with no SRPD-depression endorsement as reference)

^a^Note that because 100 % of the *N* = 144 who chronified in 2007 met criteria for severe average headache pain, the OR was inestimable and therefore the *p* value was set to 1. This is why the upper bounds on the average headache pain rating confidence interval in Models 2–4 were so extreme

### Adjusted longitudinal modeling

To assess the predictors of new-onset CM in 2006 and 2007 in persons with EM in the preceding years, the two-stage transition model was conditioned on a series of lagged and un-lagged predictors. Depression was the predictor of primary interest in this study and remained significant in all models (Table 2). After adjusting for sociodemographic features (Model 1), the odds of transformation were elevated in persons with depression (OR = 3.22; 95 % CI 1.65–6.25). Under Model 2, which adds to Model 1 adjustments for cutaneous allodynia, SRPD-anxiety, migraine pain intensity, monthly headache days, migraine symptom severity, use of anti-depressants, and medication overuse, the effect of depression on new-onset CM remained robust and significant (OR = 1.65, 95 % CI 1.12–2.45) (for intermediate models, see Webtable 1).

**Table 2:** Multivariate predictors of chronic migraine onset in persons with EM the year prior to onset

Predictive factors	Model 1 OR (95 % CI)^a^	Model 2 OR (95 % CI)^b^
Age	0.99 (0.98–1.01)	1.00 (0.99–1.01)
Gender	0.75 (0.37–1.51)	1.03 (0.71–1.50)
Income	0.90 (0.74–1.09)	0.85 (0.76–0.95)*
Insurance	0.82 (0.41–1.61)	0.89 (0.61–1.29)
BMI (linear)	0.91 (0.80–1.04)	0.91 (0.85–0.96)*
BMI (quadratic)	1.00 (1.00–1.00)	1.00 (1.00–1.00)*
Cutaneous allodynia		1.04 (0.76–1.41)
SRPD-anxiety		1.31 (0.94–1.84)
Pain intensity (≥4)		2.18 (0.67–7.13)
Headache frequency (days/month)		1.29 (1.21–1.36)*
Migraine symptom score		1.06 (1.01–1.11)*
Anti-depressant use		1.35 (0.95–1.91)
Medication overuse		1.79 (1.26–2.54)*
Depression (PHQ-9)^c^	3.22 (1.65–6.25)*	1.65 (1.12–2.45)*

Values are OR, 95 % CI. *N* = 304 (2.27 %) events out of 13,372 trials and 97.73 % non-events for both models

* Indicates that data are significant at the *p* < 0.05 level or below

^a^Model 1: adjusted for age (continuous), gender (binary, reference = male), income (linear trend in cumulative categories), health insurance status (binary, reference = uninsured), and BMI (continuous and quadratic)

^b^Model 2: adjusted for sociodemographics and for cutaneous allodynia (binary, diagnosis defined as score >3, SRPD-anxiety (binary, with no SRPD-anxiety endorsement as reference), anti-depressant use (binary, reference = no use), medication overuse proxy (binary, reference = no overuse), headache pain intensity (binary, no/mild pain (scores 0–3) versus combination of moderate (scores 4–6), moderately severe (scores 7–8), severe (scores 9–10), migraine symptom score (continuous), and headache frequency (headache days/month)

^c^Depression (PHQ-9) = dichotomous definition defined by a PHQ-9 cut score ≥15

**Fig. 3:**
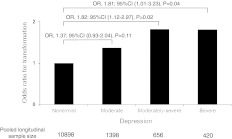
Dose–response relationship between severity of depression and risk of developing new-onset CM

To assess the influence of depression severity on risk of transformation, we ran two models using PHQ-9-based measures of depression severity as predictors of transformation. When omitting headache frequency and SRPD-depression but adjusting for sociodemographics, cutaneous allodynia, SRPD-anxiety, and migraine pain intensity under Model 3 moderate (OR = 1.77, 95 % CI 1.25–2.52), moderately severe (OR = 2.35, 95 % CI 1.53–3.62), and severe depression (OR = 2.53, 95 % CI 1.52–4.21) all significantly differed from none/mild depression in rates of transformation. The addition of the headache days per month covariate in Model 4 attenuated, but did not eliminate, the dose-response effects on transformation for moderately severe (OR = 1.82, 95 % CI 1.12–2.97), and severe depression (OR = 1.81, 95 % CI 1.01–3.23) compared to none/mild depression. Full details for dose–response models are given in Table 3. Though elevated depression remained significantly associated with transformation in Model 4, the dose-response effect appeared to asymptote once moderately severe depression manifested, with no difference in point estimates observed between effects for moderately severe and severe depression (see Fig. 3).

**Table 3:** Predictors of CM onset based on depression severity and SRPD-depression

Predictors	Model 3 OR (95 % CI)	Model 4 OR (95 % CI)
Age	1.00 (0.99–1.01)	1.00 (0.99–1.01)
Gender (female)	0.92 (0.65–1.29)	1.01 (0.70–1.46)
Income	0.85 (0.77–0.94)*	0.86 (0.77–0.96)*
BMI (linear)	0.92 (0.87–0.97)*	0.90 (0.85–0.96)*
BMI (quadratic)	1.00 (1.00–1.00)*	1.00 (1.00–1.00)*
Cutaneous allodynia	1.45 (1.11–1.91)*	1.20 (0.89–1.61)
SRPD-anxiety	1.40 (1.04–1.88)*	1.21 (0.86–1.72)
Pain intensity (≥4)	2.96 (0.94–9.27)	2.39 (0.73–7.78)
Headache frequency		1.29 (1.22–1.37)*
Depression: moderate versus none/mild	1.77 (1.25–2.52)*	1.37 (0.93–2.04)
Depression: moderately severe versus none/mild	2.35 (1.53–3.62)*	1.82 (1.12–2.97)*
Depression: severe versus none/mild	2.53 (1.52–4.21)*	1.81 (1.01–3.23)*
SRPD-depression^a^		1.38 (0.98–1.93)

Values are OR, 95 % CI. *N* = 304 (2.27 %) events out of 13,372 trials and 97.73 % non-events for both models

All models adjusted for age (continuous), gender (binary, reference = male), income (linear trend in cumulative categories), BMI (continuous and quadratic), health insurance status (binary, reference = uninsured), cutaneous allodynia (binary, diagnosis defined as score >3), SRPD-anxiety (binary, with no SRPD-anxiety as reference), and average headache pain intensity [no/mild pain (scores 0–3) versus combination of moderate (scores 4–6), moderately severe (scores 7–8), and severe (scores 9–10)]. In addition, the final model was adjusted for headache days per month preceding transformation to CM

Depression-PHQ-9 (categorical)

* Indicate data are significant at the *p* < 0.05 level or below

^a^SRPD-depression: (binary, with no SRPD-depression endorsement as reference)

## Discussion

This study demonstrates that in persons with EM, depression for 1 year is associated with the new onset of CM in the next year. Depression, as measured by the PHQ-9, remained a significant predictor of transformation after adjusting for sociodemographic variables, headache features including monthly headache day frequency, other comorbidities and medication use. In addition, analyses probing the dose–response effect of depression demonstrated that the risk of CM onset increased with the severity of depression. Though there is a large literature demonstrating that migraine and depression are comorbid and bidirectionally linked [10, 12, 20, 21], the influence of depression on the clinical course of migraine has rarely been investigated. We are not aware of comparable longitudinal population-based studies that assess the role of depression as a risk factor for the transformation of EM to CM. In evaluating this association, we endeavored to identify a parsimonious set of potential confounders and effect modifiers. We assessed candidate covariates based on previous population-based reports [4, 5, 22, 23] and univariate analyses in this sample.

In addition to depression, BMI, anxiety, several headache features (including headache frequency, average pain intensity, migraine symptom severity score, and allodynia) and medication overuse all had univariate associations with CM onset (Table 1). We also examined sociodemographic variables including age, marital status and race as well as medical covariates (SRPD-diabetes, SRPD-hypertension and smoking), previously associated with migraine prognosis [4, 22, 24]; these sociodemographic and medical covariates were not predictors of CM onset in the current study. Though alcohol consumption and smoking are associated with depression [25, 26], they were not associated with CM onset and were not included in our final models. In a longitudinal model adjusting for sociodemographic covariates only (Model 1), depression was associated with a threefold increased risk of CM onset. In the fully adjusted model (Model 2), the effect of depression on CM onset, though highly significant, was substantially attenuated. Along with depression, headache frequency, migraine symptom severity and medication overuse remained significant predictors of CM onset. Because the relationships among covariates are complex, we ran a series of nested models presented in the Webtable. The effects of allodynia and BMI vary in these models, depending upon the other covariates.

We also assessed the influence of treatment on progression to CM from EM. Medication overuse was a significant risk factor for development of CM, a finding compatible with earlier studies [4, 5, 23, 27]. Use of antidepressants was not significantly associated with risk of CM onset in our data; however, confounding by indication may contribute to this result. That is, persons selected for antidepressants may be at higher risk for progression prior to treatment by virtue of the association between depression (or factors associated with depression) and CM onset. Antidepressant pharmacotherapy, employed in both depression treatment and migraine prophylaxis, may reduce the risk of progression through an effect on both headache frequency and depression.

Though our findings indicate that persons with EM and depression develop CM at increased rates, the causal nature of this association remains uncertain. The process of CM onset is likely heterogeneous. We propose three hypotheses to account for the linkage between depression and the onset of CM based on the approach suggested by Lipton and Silberstein [28]: (1) depression may directly contribute to the onset of CM, (2) depression may arise as a consequence of escalating migraine frequency, and (3) CM and depression may share genetic or environmental risk factors that contribute to this association. These hypotheses should not be considered mutually exclusive as more than one of them may be at least partially correct.

Under the first hypothesis, depression is involved in an as yet to be determined causal path which increases the probability that persons with EM will progress to CM. This possibility is supported by the development of depression prior to the onset of CM, the depression-dose effect and the robustness of the findings despite adjustment for many potential confounders. In addition, the association has a biologically plausible foundation based on central sensitization, as discussed below [29].

The relationship between cutaneous allodynia, a marker of central sensitization, and depression has been addressed in experimental animal and human pain studies [30, 31]. Animal studies [31] have also shown that depression may induce hyperalgesia, another marker of central sensitization. Patients with migraine and chronic tension-type headaches have previously been reported to have muscular and cutaneous hyperalgesia [32, 33]. Given the strong association between depression and allodynia [33], depression and CM [10] and the higher prevalence of allodynia in CM [33], it is possible that depression facilitates the development of allodynia in EM which in turn lowers the threshold for headache onset, increasing the number of headache days [18]. In further support of this mechanism, functional neuroimaging studies in clinical chronic and experimental pain have demonstrated neuroplastic changes in the anterior cingulate cortex and the amygdala [34]. Depression and anxiety are also associated with increased activity in the amygdala which may contribute to the activation of pain-facilitating pathways [35]. In combination, these findings support the hypothesis that depression could cause CM onset through effects on central sensitization.

Appropriate randomized trials to support this model have not been conducted. In such a trial, one could treat patients with EM and depression with an antidepressant that did not influence headache (e.g., a selective serotonin reuptake inhibitor or bupropion), predicting that treating depression should prevent CM onset. The credibility of inferences under such a design depends upon convincing evidence that the treatment influences depression but not migraine.

Under the second mechanism, depression may arise as a consequence of more severe or escalating disease. The assumption here is that escalating attack frequency, below the level needed to meet a CM definition, may contribute to the development of depression. Escalating headache frequency could reduce self-efficacy creating depression through the mechanism of learned helplessness [36]. For inferential tests of this hypothesis to be valid, treatments employed in an experimental design would be required to effectively treat CM but not directly influence depression. In a small, open label study, researchers demonstrated that treating CM with onabotulinumtoxinA resulted not only in reductions of headache frequency, but also improvements in depression and anxiety, thus demonstrating that reducing headache frequency, without treating depression directly, leads to improvement in psychological outcomes [37]. Larger scale, randomized trial data could provide much stronger support for this hypothesis.

Under our third hypothesis, CM and depression are linked by shared genetic or environmental risk factors. For example, variants in catecholamine genes could predispose people to both CM and depression and account for the linkage between them [38]. Alternatively, a persistently stressful environment could contribute to both the development of CM and depression [39]. Chronic stress is a well-recognized risk factor for both depression and CM [39]. Specifically, Rivat et al. [40] found that chronic stress, a common precursor to depression as outlined above, increases the expression of genes governing the iNOS and COX-2 inflammatory molecules and induces neuroinflammatory conditions suppressing the mechanical nociceptive threshold thereby increasing hypersensitivity and hyperalgesia. Given accurate measures of any of these possible mechanisms, mediation models could be examined in which the association between depression and CM onset could be tested for robustness to the intervening effect of potential shared genetic or environmental variables.

These hypotheses seem to be reasonable explanations for the association between depression and CM onset observed in this study. The experimental designs and analyses described, if undertaken, would serve to elucidate the relative strength of each possible mechanism discussed.

This study has a number of strengths. We evaluated a large population-based sample, followed participants longitudinally and systematically assessed both migraine and depression using well-validated instruments. The AMPP study diagnostic module is validated for the diagnosis of migraine in the population [14]. In addition, our measure of depression was based on the validated PHQ-9 assessment.

There are a number of limitations in our study. Assessments of migraine and depression status were based on self-reported validated questionnaires and not in-person interviews or review of medical data. Moreover, anxiety was assessed by self-report of having received a diagnosis from a healthcare professional. Misclassification of exposures and outcomes is possible. For example, we included respondents meeting criteria for chronic tension-type headache in the control group. While a legitimate transition state for those with EM in a preceding year, the inclusion of chronic tension-type headache in the reference group could attenuate the degree of association between depression and transformation. However, our findings were robust, and this effect is likely very small due to the modest number of chronic tension-type headache respondents observed in the reference groups for the two transformation events (*N* = 14 and *N* = 28, respectively). Therefore, the impact of the inclusion of chronic tension-type headache in the reference is likely small.

This longitudinal population-based study demonstrates the association between depression and the onset of CM. Given that this is an observational study, we cannot determine what role depression plays linking EM to CM. However, given the strong association between depression and CM onset observed here, for the sake of patients routine screening for depression should be considered, if for no other reason than that it is a strong comorbidity with CM onset and a complicating factor for treatment. In addition, because we do not, as yet, have a biomarker useful for identifying patients at risk for CM onset, given the predictive strength of the covariates as risk factors for CM onset, clinicians can routinely screen for depression, allodynia, headache frequency, migraine symptom severity, and medication overuse, as markers to improve detection of and modify treatment for at-risk patients. Future work will focus on appropriately designed trials to elucidate the operating mechanism linking EM, depression, and CM onset.

## Electronic supplementary material

Below is the link to the electronic supplementary material.

Supplementary material 1 (DOC 74 kb)
