# Supplementary material for: Depression and risk of transformation of episodic to chronic migraine
Source: J Headache Pain. 2012 Sep 25;13(8):615–24. doi: 10.1007/s10194-012-0479-9 (PMC3484253; doi:10.1007/s10194-012-0479-9)
Supplement: Supplementary file 1 — Supplementary material 1 (DOC 74 kb) [file 10194_2012_479_MOESM1_ESM.doc]

**Title: Depression and risk of transformation of episodic to chronic migraine**

**Web Tables**

| **Webtable 1.** Multivariate predictors of Chronic Migraine Onset in Persons with EM the year prior to onset | | | | |
| --- | --- | --- | --- | --- |
| **Predictive factors** | **Model 1**  **OR (95% CI)** | **Model 2**  **OR (95% CI)** | **Model 3**  **OR (95% CI)** | **Model 4**  **OR (95% CI)** |
| Age | 0.99 (0.98 - 1.01) | 1.00 (0.99-1.01) | 1.00 (0.99-1.01) | 1.00(0.99-1.01) |
| Gender | 0.75 (0.37 - 1.51) | 0.92 (0.65-1.28) | 1.04 (0.71-1.50) | 1.03(0.71-1.50) |
| Income | 0.90 (0.74 - 1.09) | 0.85 (0.77-0.94)* | 0.85 (0.75-0.95)* | 0.85(0.76-0.95)* |
| Insurance | 0.82 (0.41 - 1.61) | 0.91 (0.65-1.27) | 0.90 (0.62-1.30) | 0.89(0.61-1.29) |
| BMI (linear) | 0.91 (0.80 - 1.04) | 0.92 (0.87-0.97)* | 0.91 (0.85-0.97)* | 0.91(0.85-0.96)* |
| BMI (quadratic) | 1.00 (1.00-1.00) | 1.00 (1.00-1.00)* | 1.00 (1.00-1.00)* | 1.00(1.00-1.00)* |
| Cutaneous allodynia |  | 1.41 (1.08-1.83)* | 1.07 (0.79-1.46) | 1.04(0.76-1.41) |
| SRPD-Anxiety |  | 1.53 (1.15-2.04)* | 1.44 (1.03-2.03) | 1.31(0.94-1.84) |
| Pain intensity (4+) |  |  | 2.15 (0.66-6.98) | 2.18(0.67-7.13) |
| Headache frequency (days/month) |  |  | 1.30 (1.21-1.39)* | 1.29(1.21-1.36)* |
| Migraine symptom score |  |  | 1.07 (1.02-1.12)* | 1.06(1.01-1.11)* |
| Anti-depressant use |  |  |  | 1.35(0.95-1.91) |
| Medication overuse |  |  |  | 1.79(1.26-2.54)* |
| Depression (PHQ-9) | 3.22 (1.65 - 6.25)* | 2.01 (1.42-2.85)* | 1.74 (1.18-2.59)* | 1.65(1.12-2.45)* |
| - Values are OR, 95% CI. - * indicates that data are significant at the *p*<0.05 level or below - Model 1: Adjusted for age (continuous), gender (binary, reference=male), income (linear trend in cumulative categories), health insurance status (binary, reference=uninsured), and BMI (continuous and quadratic). - Model 2: Adjusted for cutaneous allodynia (binary, diagnosis defined as score > 3) and SRPD-anxiety (binary, with no SRPD-anxiety endorsement as reference). - Model 3: Adjusted for headache pain intensity (binary, no/mild pain (scores 0-3) vs. combination of moderate (scores 4-6), moderately severe (scores 7-8), severe (scores 9-10), migraine symptom score (continuous), and headache frequency (headache days/month) - Model 4: Adjusted for anti-depressant use (binary, reference=no use) and medication overuse proxy (binary, reference=no overuse) - Depression (PHQ-9)= dichotomous definition defined by a PHQ-9 cut-score ≥15 | | | | |

| **Webtable 2.** Predictors of CM onset based on Depression Severity and SRPD-Depression. | | | |
| --- | --- | --- | --- |
| **Predictors** | **Model 5**  **OR (95% CI)** | **Model 6**  **OR (95% CI)** | **Model 7**  **OR (95% CI)** |
| Age | 1.00 (0.99 - 1.01) | 1.00 (0.99 - 1.01) | 1.00 (0.99 - 1.01) |
| Gender (female) | 0.92 (0.65 - 1.29) | 0.92 (0.65 - 1.30) | 1.01 (0.70 - 1.46) |
| Income | 0.85 (0.77 - 0.94)* | 0.86 (0.78 - 0.94) * | 0.86 (0.77 - 0.96)* |
| BMI (linear) | 0.92 (0.87 - 0.97)* | 0.92 (0.87 - 0.97) * | 0.90 (0.85 - 0.96)* |
| BMI (quadratic) | 1.00 (1.00 - 1.00)* | 1.00 (1.00 - 1.00)* | 1.00 (1.00 - 1.00)* |
| Cutaneous allodynia | 1.45 (1.11 - 1.91)* | 1.44 (1.10 - 1.90) * | 1.20 (0.89 - 1.61) |
| SRPD-Anxiety | 1.40 (1.04 - 1.88)* | 1.22 (0.88 - 1.68) | 1.21 (0.86 - 1.72) |
| Pain Intensity (4+) | 2.96 (0.94 - 9.27) | 2.91 (0.93 - 9.13) | 2.39 (0.73 - 7.78) |
| Headache frequency |  |  | 1.29 (1.22 - 1.37)* |
| Depression: Moderate vs. None/mild | 1.77 (1.25 - 2.52)* | 1.65 (1.15 - 2.36) * | 1.37 (0.93 - 2.04) |
| Depression: Moderately-severe vs. None/mild | 2.35 (1.53 - 3.62)* | 2.13 (1.37 - 3.31) * | 1.82 (1.12 - 2.97)* |
| Depression: Severe vs. None/mild | 2.53 (1.52 - 4.21)* | 2.28 (1.36 - 3.82) * | 1.81 (1.01 - 3.23)* |
| SRPD-Depression |  | 1.39 (1.02 - 1.88)* | 1.38 (0.98 - 1.93) |

- Values are OR, 95% CI. * indicate data are significant at the *p*<0.05 level or below
- All models Adjusted for age (continuous), gender (binary, reference=male), income (linear trend in cumulative categories), BMI (continuous and quadratic), health insurance status (binary, reference=uninsured), cutaneous allodynia (binary, diagnosis defined as score > 3), SRPD-anxiety (binary, with no SRPD-anxiety as reference), and Average headache pain intensity (no/mild pain (scores 0-3) vs. combination of moderate (scores 4-6), moderately severe (scores 7-8), and severe (scores 9-10)). In addition, the final model was adjusted for headache days per month preceding transformation to CM.
- Depression- PHQ-9 (categorical).
- SRPD-Depression: (binary, with no SRPD-depression endorsement as reference).
